# Complete plastid genome of *Eriobotrya japonica* (Thunb.) Lindl and comparative analysis in Rosaceae

**DOI:** 10.1186/s40064-016-3702-3

**Published:** 2016-11-29

**Authors:** Liqun Shen, Qijie Guan, Awais Amin, Wei Zhu, Mengzhu Li, Ximin Li, Lin Zhang, Jingkui Tian

**Affiliations:** 1 Department of Biomedical Engineering, Zhejiang University, Hangzhou, 310027 China; 2 Changshu Qiushi Technology Co. Ltd., Changshu, 215500 China

**Keywords:** *Eriobotrya*, Loquat, Chloroplast genome, Rosaceae, Gene evolution

## Abstract

*Eriobotrya japonica* (Thunb.) Lindl (loquat) is an evergreen Rosaceae fruit tree widely distributed in subtropical regions. Its leaves are considered as traditional Chinese medicine and are of high medical value especially for cough and emesis. Thus, we sequenced the complete plastid genome of *E. japonica* to better utilize this important species. The complete plastid genome of *E. japonica* is 159,137 bp in length, which contains a typical quadripartite structure with a pair of inverted repeats (IR, 26,326 bp) separated by large (LSC, 89,202 bp) and small (SSC, 19,283 bp) single-copy regions. The *E. japonica* plastid genome encodes 112 unique genes which consist of 78 protein-coding genes, 30 tRNA genes and 4 rRNA genes. Gene structure and content of *E. japonica* plastid genome are quite conserved and show similarity among Rosaceous species. Five large indels are unique to *E. japonica* in comparison with *Pyrus pyrifolia* and *Prunus persica*, which could be utilized as molecular markers. A total of 72 simple sequence repeats (SSRs) were detected and most of them are mononucleotide repeats composed of A or T, indicating a strong A or T bias for base composition. The Ka and Ks ratios of most genes are lower than 1, which suggests that most genes are under purifying selection. The phylogenetic analysis described the evolutionary relationship within Rosaceae and fully supported a close relationship between *E. japonica* and *P. pyrifolia.*

**Electronic supplementary material:**

The online version of this article (doi:10.1186/s40064-016-3702-3) contains supplementary material, which is available to authorized users.

## Background

Chloroplast (cp), which is derived from free-living cyanobacteria through endosymbiosis (Keeling 2004), plays an essential role in photosynthesis and many biosynthetic activities such as biosynthesis of certain amino acids and fatty acids. Chloroplast contains its own genome which displays a typical quadripartite structure with two copies of inverted repeats separated by large single copy and small single copy (Nguyen et al. 2015). In general, plastomes of angiosperms range from 120 to 170 kb and mostly contain 100–120 different genes. The chloroplast genome is usually recognized as highly conserved in gene structure and content, especially in closely related groups. However, IR loss or expansion/contraction which contributes to the variation in genome size can be easily found in some clades. Gymnosperms such as *Taxus chinensis var. mairei* (Zhang et al. 2014b) and *Cephalotaxus oliveri* (Yi et al. 2013) were identified to lose one copy of IR, leading to a decrease in genome size. Ma et al. (2013) found a significant IR expansion in *Mahonia bealei* in which 15 genes had an additional duplication in IR regions. Slight shift of IR/SC boundaries appears more commonly and may cause small changes in the extent of IR (Goulding et al. 1996). Consequently, structure differences of IR have been considered as important features to provide insights into gene evolution among species.

The plastid genome is good resource to provide sufficient information for phylogenetic analysis and DNA barcoding. Thanks to rapid development of next-generation sequencing, the number of whole plastid genome available is increasing constantly, which makes large-scale phylogenetic research based on plastid genomes possible (Jansen et al. 2007). In addition, comparative analysis among species can provide large amount of genetic information such as insertion or deletion (indel) and nucleotide substitutions, which can be utilized for diversity analysis and molecular markers (Cho et al. 2015).

There are over 100 genera containing approximately 3000 species in Rosaceae which is medium-sized, but of high economic importance (Potter et al. 2007). As the third most important economical family in temperate regions, many edible fruits like apple (*Malus*), loquat (*Eriobotrya*), pear (*Pyrus*) and peach (*Prunus*) as well as ornamental plants such as rose (*Rosa*) are included. However, till now, phylogenetic relationships within Rosaceae family have been uncertain. Traditional morphological studies divided Rosaceae into 4 subfamilies including Spiraeoideae, Maloideae, Rosoideae and Prunoideae according to the type of fruit (Kalkman 1988). Molecular studies however, put forward different opinions that Rosaceae was divided into three subfamilies: Dryadoideae, Rosoideae and Spiraeoideae (Potter et al. 2007). As the plastid genome can offer useful phylogenetic information, several plastomes of Rosaceae plants have been sequenced and reported, such as *Malus domestic* (http://www.rosaceae.org/projects/apple_genome), *Pyrus pyriofolia* (AP012207.1), *Prunus persica* (HQ336405) and *Fragaria virginiana* (NC_019602). Most of these studies focused on the comparative analysis and evolutionary studies within genera. Wang et al. (2013) reported the chloroplast genome of *Prinsepia utilis* and reconstructed phylogenetic relationships within Rosaceae but with low bootstrap values. So more closely related plastid genomes are needed to deal with intrafamilial classifications within Rosaceae.

*Eriobotrya japonica* (Thunb.) Lindl (loquat), which belongs to Rosaceae, is widely distributed in temperate and subtropical zones of Asian, European and American countries (Gisbert et al. 2009). *Eriobotrya japonica* originated in China and later was introduced to Mediterranean basin, Japan and Florida (Blasco et al. 2014). *Eriobotrya japonica* is an important economic fruit crop with high edible, medical as well as ornamental values. The fruit of *E. japonica* is greatly appreciated by consumers due to its good taste and high nutrition values. Its leaves, where pharmaceutically active compounds proved to be found (Sharpe 2010), have long been considered as one kind of traditional Chinese medicine. Due to natural multiplication and artificial cultivation for long time, genetic variation can be easily found within loquat which makes the identification and classification of loquat more difficult. Molecular markers such as RAPD, AFLP or SSRs have been rapidly developed these years and applied into loquat studies. Vilanova et al. (2001) applied RAPD technology into 33 loquat cultivars for identification from different regions. Soriano et al. (2005) first demonstrated the usefulness of SSRs markers in genetic analysis and identification in *E. japonica*. Many researches utilized AFLP and SSRs markers to investigate genetic variation, diversity and identification among loquat accessions (He et al. 2011; Fukuda et al. 2013; Blasco et al. 2014), which provided information for diversity analysis and plant breeding program. However, few molecular markers based on plastomes have been developed since completed plastid genome of *E. japonica* is not available, lagging behind other species of Rosaceae.

In this study, we report the first complete plastid genome of *E. japonica* using next-generation sequencing method (Illumina Hiseq 2000) and conduct comparative analysis with other Rosaceous species, *P. pyrifolia* and *P. persica* in particular, which will not only help with species identification or germplasm selection but also provide insights into phylogenetic evolution of Rosaceae family.

## Methods

### Taxon sampling, DNA sequencing and genome assembly

Fresh leaves of *Eriobotrya japonica* were collected from the nursery garden in Zhejiang University to prepare for isolation of chloroplast DNA. The two-step Percoll gradient method (Aronsson and Jarvis 2002) was used to isolate purified *E. japonica* chloroplasts. The chloroplast DNA was then extracted and purified using the CTAB method (He 2011) with slight modifications. The whole chloroplast library was constructed by 5 μg purified cp DNA. The cp genome of *E. japonica* was then sequenced using Illumina Hiseq 2000 system, which generated in total 4,484,369 raw pair-end reads for this project. As many low-quality reads were included in the generated raw reads, SolexaQA v1.0 (Cox et al. 2010) was used to filter low-quality reads with the settings-h 27 and-l 60. Then 6050 contigs with an average length of 206 bp were generated by Soapdenovo v1.3 (Luo et al. 2012) with Kmer size equal to 59 bp. The similar strategy with Zhang et al. (2014b) was then used for the assembly. The complete plastid genome of *Pyrus pyrifolia* (AP012207) extracted from NCBI (http://www.ncbi.nlm.nih.gov/) was selected as a reference and Blastz (Schwartz et al. 2003) perl script was used to map all contigs to the reference in order to determine the order and direction of these contigs. The following steps was taken repeatedly to fill up the gaps: first, BLAT v.34 (Kent 2002) was used to map raw sequence reads to both ends of the assembled contigs; second, assembled contigs were extended by connecting up with overlapping reads which were best overlapped with the contigs (Zhang et al. 2014b). For the gaps remaining between contigs, we designed six pairs of primers for PCR amplification to close these gaps (Additional file 1: Table S1). Also, to avoid mistakes during our assembly, another four primers were designed to confirm IR/SC boundaries (Additional file 1: Table S1). All PCR products were sequenced by conventional Sanger sequencing and the results were consistent with our assembly. Thus, the whole plastid genome of *E. japonica* was obtained.

### Genome annotation, codon usage and comparative analysis

The *Eriobotrya japonica* plastid genome was annotated using the program Dual Organellar GenoMe Annotator (Wyman et al. 2004). Start and stop codons of protein-coding genes were then manually checked and adjusted if necessary, by comparing *E. japonica* with other Rosaceae plastomes. Any genes not found by DOGMA were verified missing by the use of blastn and blastx online searches (https://blast.ncbi.nlm.nih.gov/Blast.cgi). Transfer RNA genes were identified and further confirmed with tRNAscan-SE 1.21 (Lowe and Eddy 1997) using the mito/chloroplast source setting. Then the whole record was deposited into Genbank with accession number KT633951. The physical map of the complete plastid genome was drawn by OGdraw v1.2 online tool (Lohse et al. 2007). Codon usage and relative synonymous codon usage (RSCU) analysis of all protein-coding genes were conducted using MEGA5 (Tamura et al. 2011). In order to perform identity analysis, seven Rosaceae cp genomes, *Fragaria virginiana* (NC_019602), *Pentactina rupicola* (JQ041763), *Prinsepia utilis* (NC_021455), *Prunus persica* (HQ336405), *Pyrus pyrifolia* (AP012207), and *Rosa odorata* (KF753637) were extracted from NCBI and were aligned with *E. japonica* respectively to compute pairwise identity using mVISTA program (Frazer et al. 2004). Large indels with no less than 40 bp in size were detected through pairwise alignment among *E. japonica, P. pyrifolia* and *P. persica*. For these unique large indels of *E. japonica*, five pairs of primers were also designed to avoid assembly errors (Additional file 2: Table S2).

### Repeat structure identification

Reputer online program (Kurtz et al. 2001) was used to detect forward, palindrome, reverse and complementary repeat structures with structure size greater than 20 bp and hamming distance equal to 0 (100% identity).

Simple sequence repeats (SSRs) were detected within completed genomes of *Eriobotrya. japonica* using MISA v1.0 (Thiel et al. 2003) with parameter settings of mononucleotide, dinucleotide, tri- or tetranucleotide and hexanucleotide repeats no less than 10, 12, 15 and 24 bases respectively.

### Nucleotide substitution in coding regions

All 78 functional protein-coding genes were extracted from *Eriobotrya japonica, Pyrus pyrifolia* and *Prunus persica* chloroplast genome. Each gene of *P. pyrifolia* and *P. persica* was aligned with that of *E. japonica* using clustalX v2.1 (Thompson et al. 1997). The alignment file was then put into Dnasp v5 (Librado and Rozas 2009) to calculate the synonymous (Ks) and nonsynonymous (Ka) substitution rates. Transition (Ts), transversion (Tv) and P-distance of each gene were calculated using MEGA5 (Tamura et al. 2011).

### Phylogenetic analysis

All 78 protein-coding genes extracted from the plastomes of 7 Rosaceous plants (*Fragaria virginiana*, NC_019602; *Pentactina rupicola*, JQ041763; *Prinsepia utilis*, NC_021455; *Prunus persica*, HQ336405; *Pyrus pyrifolia*, AP012207; *Rosa odorata*, KF753637 and *Eriobotrya japonica*) and *Morus indica* (NC_008359, as an outgroup) were used to construct Maximum likelihood (ML) tree and Maximum parsimony (MP) tree. All 78 protein coding genes were concatenated and then aligned using MAFFT v7 (Katoh and Standley 2013). Gblock v0.91 (Talavera and Castresana 2007) was used to select the conserved regions, which resulted in a total length of 67,104 bases for MP and ML analysis. The aligned sequences of conserved regions were then tested in DAMBE v5.3.19 (Xia and Lemey 2009) for saturation of substitution. The results revealed that Iss (index of substitution saturation) < Iss.c (the critical Iss value), indicating no substantial saturation in these sequences (Xia et al. 2003). The General Time Reversible model of substitution, incorporating invariant sites and a gamma distribution (GTR + I+G), was selected as the best model by jmodeltest2.1.7 (Posada 2008). To construct the ML tree, RaxML7.0.4 (Stamatakis 2006) was used with 1000 non-parametric replications for bootstrap settings. The base frequences estimated by RaxML was 0.305 (A), 0.174 (C), 0.203 (G), 0.317 (T) while rate matrix was 1.54, 3.07, 0.24, 0.77, 3.37, 1.00 for ac, ag, at, cg, ct, gt, respectively. PAUP^∗^4.0b10 (Swofford 2003) was used to construct MP tree with the following options implemented: Accctran was used, gaps were treated as missing, heuristic search mode used 1000 replications for bootstrap support, tree bisection-reconnection (TBR) branch-swapping, MulTrees in effect, and steepest descent off.

## Results

### Genome features of *E. japonica*

**Fig. 1:**
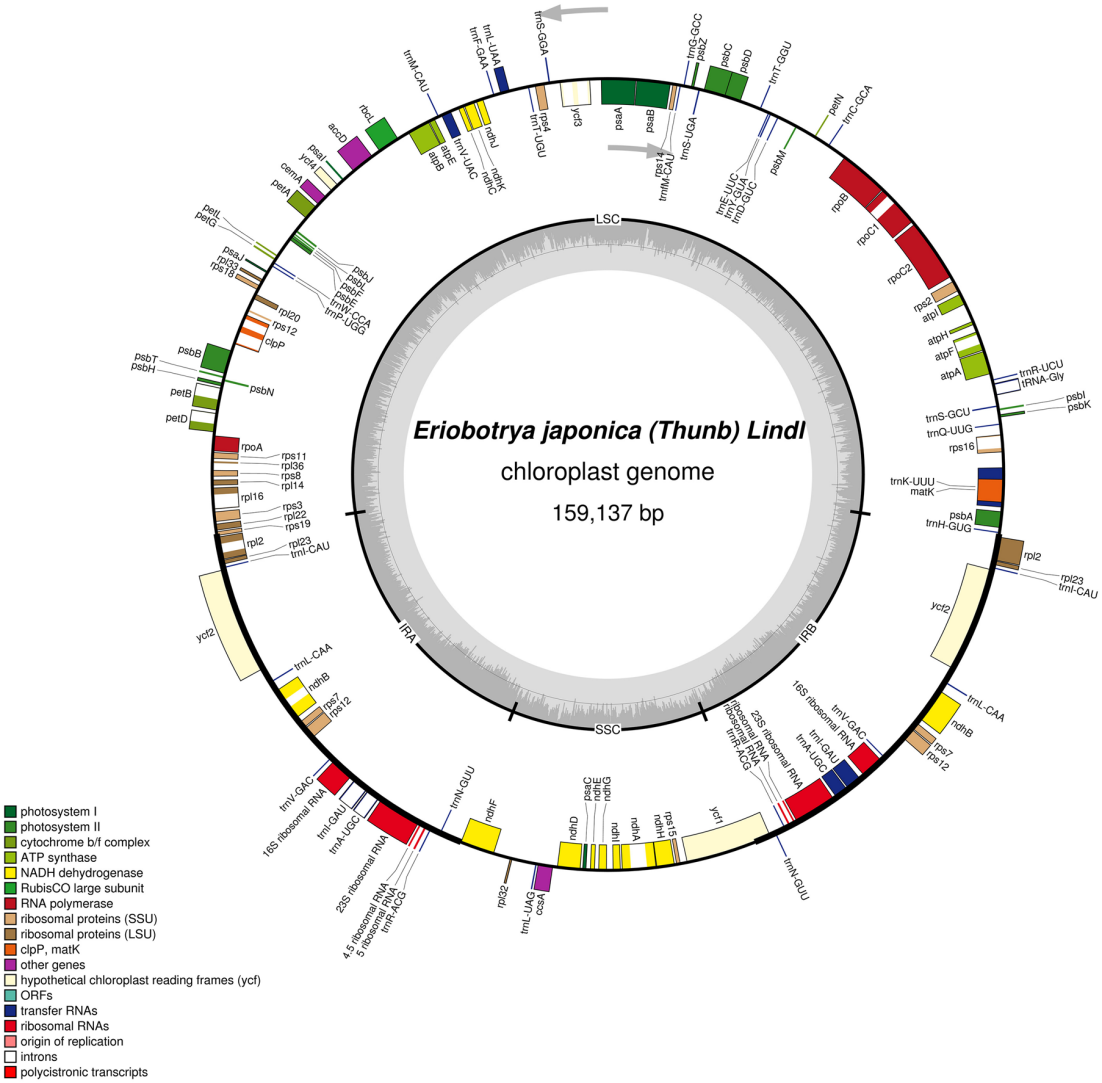
Gene map of *E. japonica* complete chloroplast genome. Exons are annotated by *coloured boxes* and introns are annotated with *white boxes*. Genes *inside* or *outside* of the *large circle* are respectively transcribed in the *clockwise* or *counterclockwise* direction. *Boundaries* of LSC, SSC, IR are annotated at *inner circle*. GC content is represented by *dark grey graph* within *inner circle*

**Fig. 2:**
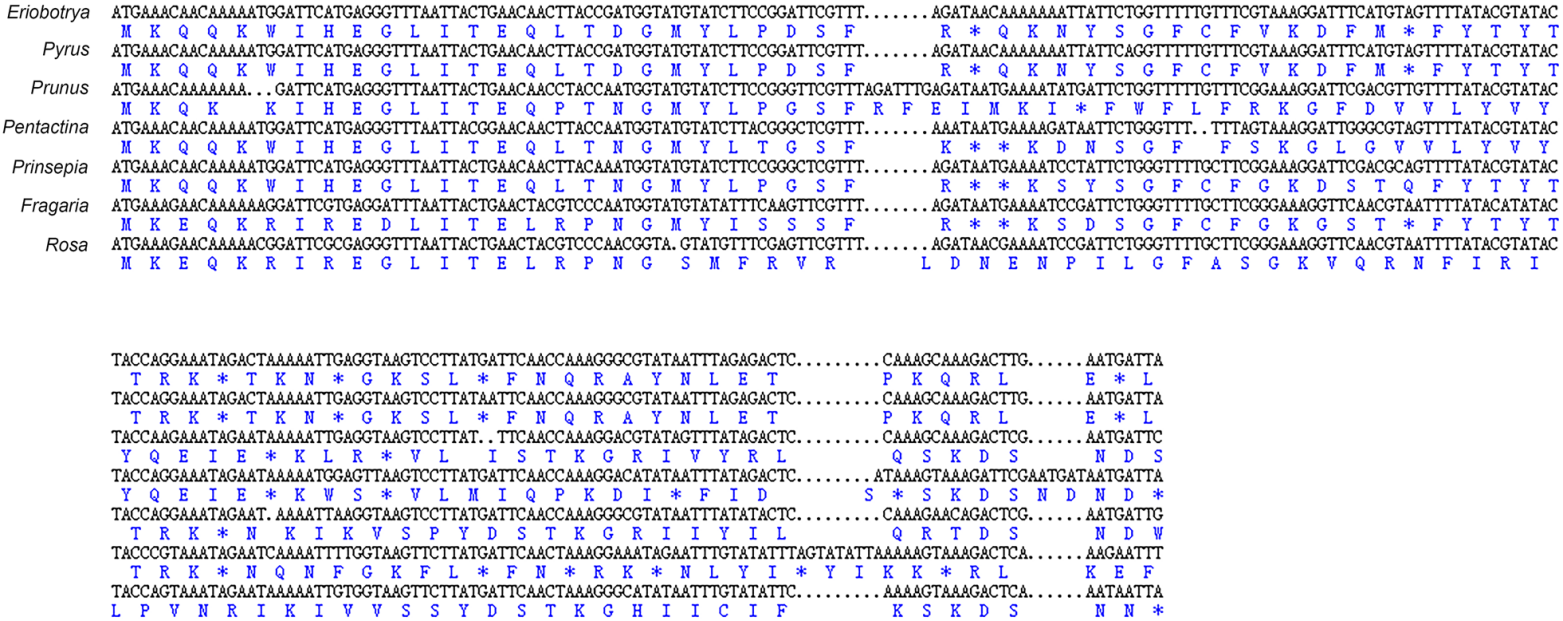
Alignment of *infA* coding regions of seven Rosaceae plastid genomes. *Asterisks* mean stop codons while *dots* mean gaps

The complete plastid genome of *Eriobotrya japonica* is 159,137 bp in length with a double-strand circle structure with a pair of IRs of 26,326 bp separated by a small single copy of 19,283 bp and a large single copy of 87,202 bp (Fig. 1). The plastid genome includes 112 functional genes in all, of which 78 are protein-coding genes, 30 are tRNA genes and 4 are rRNA genes (Table 1). Eighteen genes duplicates in IR region including 6 protein-coding genes, 7 tRNA genes and 4 rRNA genes. Eighteen genes comprising six tRNA genes contain one or two introns. Both *ycf3* and *clpP* gene contain two introns while *rps12* gene is trans-spliced with a share 5′ exon in LSC region and two 3′ exons located in IR region. Three genes, *ycf1*, *rps19* and *infA* was found as non-functional genes in the *E. japonica* plastid genome. *ycf1* and *rps19*, located in IR/SC boundary regions, became truncated as incomplete duplications of the normal copy. *infA gene* was found in *R. odorata* while it was non-functional in *E. japonica* and some other Rosaceae plastomes as several stop codons were identified in *infA* coding regions (Fig. 2).

**Table 1:** List of genes located in *E. japonica*

Group of genes	Gene names
Ribosomal RNA genes	*rrn16* ^*#*^ *,rrn23* ^*#*^ *,rrn4.5* ^*#*^ *,rrn5* ^*#*^
Transfer RNA genes	*trnA*-UGC* ^*#*^ *, trnC*-*GCA, trnD*-*GUC, trnE*-*UUC, trnF*-*GAA, trnG*-GCC*, trnG-*UCC, trnH*-*GUG, trnI*-*CAU* ^*#*^ *, trnI*-GAU* ^*#*^ *, trnK*-UUU*, trnL-*CAA* ^*#*^ *, trnL*-UAA*, trnL-*UAG, trnM*-*CAU, trnfM*-*CAU, trnN*-*GUU* ^*#*^ *, trnP*-*UGG, trnQ*-*UUG, trnR*-*UCU, trnR*-*ACG* ^*#*^ *, trnS*-*UGA, trnS*-*GCU, trnS*-*GGA, trnT*-*GGU, trnT*-*UGU, trnV*-UAC*, trnV-*GAC* ^*#*^ *, trnW*-*CCA, trnY*-*GUA*
Small subunit of ribosome	*rps2, rps3, rps4, rps7* ^*#*^ , rps8, rps11, rps12*, rps14, rps15, rps16*, rps18, rps19 ^*#*^
Large subunit of ribosome	rpl2* ^*#*^ , rpl14, rpl16*, rpl20, rpl22, rpl23 ^*#*^ *, rpl32, rpl33, rpl36*
DNA dependent RNA polymerase	rpoA, rpoB, rpoC1*, rpoC2
Subunits of photosystem I	*psaA, psaB, psaC, psaI, psaJ*
Subunits of photosystem II	*psbA, psbB, psbC, psbD, psbE, psbF, psbH, psbI, psbJ, psbK, psbL, psbM, psbN, psbT, psbZ*
Subunits of cytochrome	petA, petB*, petD*, petG, petL, petN
Subunits of ATP synthase	atpA, atpB, atpE, atpF*, atpH, atpI
ATP-dependent protease	clpP*
Large subunit of Rubisco	*rbcL*
Subunits of NADH	ndhA*, ndhB* ^*#*^ *, ndhC, ndhD, ndhE, ndhF* ^*#*^ *, ndhG, ndhH, ndhI, ndhJ, ndhK*
Maturase	*matK*
Envelop membrane protein	*cemA*
Subunit of Acetyl-CoA-carboxylase	*accD*
c-Type cytochrome synthesis gene	*ccsA*
Conserved open reading frames	*ycf1, ycf2* ^*#*^ , ycf3*, ycf4

* Gene with intron

^#^Gene duplicated in IR

Overall, 22,772 codons encoding 78 functional protein-coding genes were identified from *E. japonica* plastid genome and classified depending on codon usage (Table 2). There were 2388 codons encoding for leucine and 252 codons for cysteine, which denoted respectively the most and the least amino acids. The RSCU analysis showed a great A/T bias in the protein-coding genes, especially in the third position of the codon with G/C content at 25.9%. This pattern has been identified in former studies (Redwan et al. 2015; Tangphatsornruang et al. 2011). However, whether this pattern relates to selection for translational efficiency or nucleotide compositional biases remains unclear.

**Table 2:** Codon usage and RSCU analysis of *E. japonica* cp genome

Amino acid	Codon	No.	RSCU	tRNA	Amino acid	Codon	No.	RSCU	tRNA
Phe	UUU	850	1.34		Tyr	UAU	691	1.61	
	UUC	420	0.66	*trnF*-*GAA*		UAC	166	0.39	*trnY*-*GUA*
Leu	UUA	814	2.05	*trnL*-*UAA*	TER	UAA	43	1.65	
	UUG	485	1.22	*trnL*-*CAA*		UAG	19	0.73	
	CUU	495	1.24		His	CAU	414	1.54	
	CUC	145	0.36			CAC	123	0.46	*trnH*-*GUG*
	CUA	305	0.77	*trnL*-*UAG*	Gln	CAA	634	1.55	*trnQ*-*UUG*
	CUG	144	0.36			CAG	182	0.45	
Ile	AUU	987	1.50		Asn	AAU	834	1.55	
	AUC	364	0.55	*trnI*-*GAU*		AAC	244	0.45	*trnN*-*GUU*
	AUA	622	0.95		Lys	AAA	904	1.53	*trnK*-*UUU*
Met	AUG	537	1.00	*trnfM*-*CAU*		AAG	281	0.47	
Val	GUU	472	1.47		Asp	GAU	743	1.62	
	GUC	134	0.42	*trnV*-*GAC*		GAC	175	0.38	*trnD*-*GUC*
	GUA	504	1.57	*trnV*-*UAC*	Glu	GAA	897	1.50	*trnE*-*UUC*
	GUG	173	0.54			GAG	299	0.50	
Ser	UCU	485	1.71		Cys	UGU	190	1.51	
	UCC	259	0.92	*trnS*-*GGA*		UGC	62	0.49	*trnC*-*GCA*
	UCA	327	1.15	*trnS*-*UGA*	TER	UGA	16	0.62	
	UCG	155	0.55		Trp	UGG	396	1.00	*trnW*-*CCA*
Pro	CCU	363	1.56		Arg	CGU	301	1.33	*trnR*-*ACG*
	CCC	172	0.74			CGC	97	0.43	
	CCA	268	1.15	*trnP*-*UGG*		CGA	312	1.38	
	CCG	130	0.55			CGG	99	0.44	
Thr	ACU	483	1.64		Ser	AGU	364	1.29	
	ACC	211	0.72	*trnT*-*GGU*		AGC	109	0.39	
	ACA	363	1.23	*trnT*-*UGU*	Arg	AGA	407	1.80	*trnS*-*GCU*
	ACG	123	0.41			AGG	138	0.61	
Ala	GCU	590	1.85		Gly	GGU	528	1.35	
	GCC	194	0.61			GGC	166	0.42	*trnG*-*GCC*
	GCA	349	1.10	*trnA*-*UGC*		GGA	620	1.58	*trnG*-*UCC*
	GCG	141	0.44			GGG	254	0.65	

### Comparison with other plastid genomes in Rosaceae

Structure and content of *Eriobotrya japonica* plastid genome are conserved and share similar features with other Rosaceae plastid genomes (Table 3). The average length of six cp genomes is 157,619 bp. Among these seven Rosaceae plants, *Pyrus pyrifolia* is the largest in size at 159,922 bp while *Fragaria virginiana* is shortest at 155,621 bp. *Eriobotrya japonica* ranks following *P. pyrifolia* with 159,137 bp. *P. pyrifolia* contains the longest LSC and IR, which are 87,901 and 26,392 respectively. The SSC size of *E. japonica* is the largest, which is 46 bp longer than *P. pyrifolia*.

**Table 3:** Summary of seven Rosaceae plastid genome features

	*E. japonica*	*P. pyrifolia*	*P. rupicola*	*P. persica*	*F. virginiana*	*P. utilis*	*R. odorata*
Accession	KT633951	NC_015996	NC_016921	NC_014697	NC_019602	NC_021455	KF753637
Length	159,137	***159,922***	156,612	157,790	*155,621*	156,328	156,634
LSC	87,202	***87,901***	*84,970*	85,968	85,586	85,239	85,767
SSC	***19,283***	19,237	18,941	19,060	*18,145*	18,485	18,761
IR	26,326	***26,392***	26,351	26,381	*25,945*	26,302	26053
GC% overall	36.7	*36.6*	36.8	36.8	***37.2***	36.9	**37.2**
in LSC	34.5	*34.3*	34.6	34.6	35.1	34.7	**35.1**
in IR	42.7	42.7	*42.6*	42.6	***42.8***	42.7	42.7
in SSC	*30.3*	30.4	30.6	30.6	31.1	30.5	**31.2**

Number in bold italics indicates the largest value of line

Number in italics indicates the smallest value of line

**Fig. 3:**
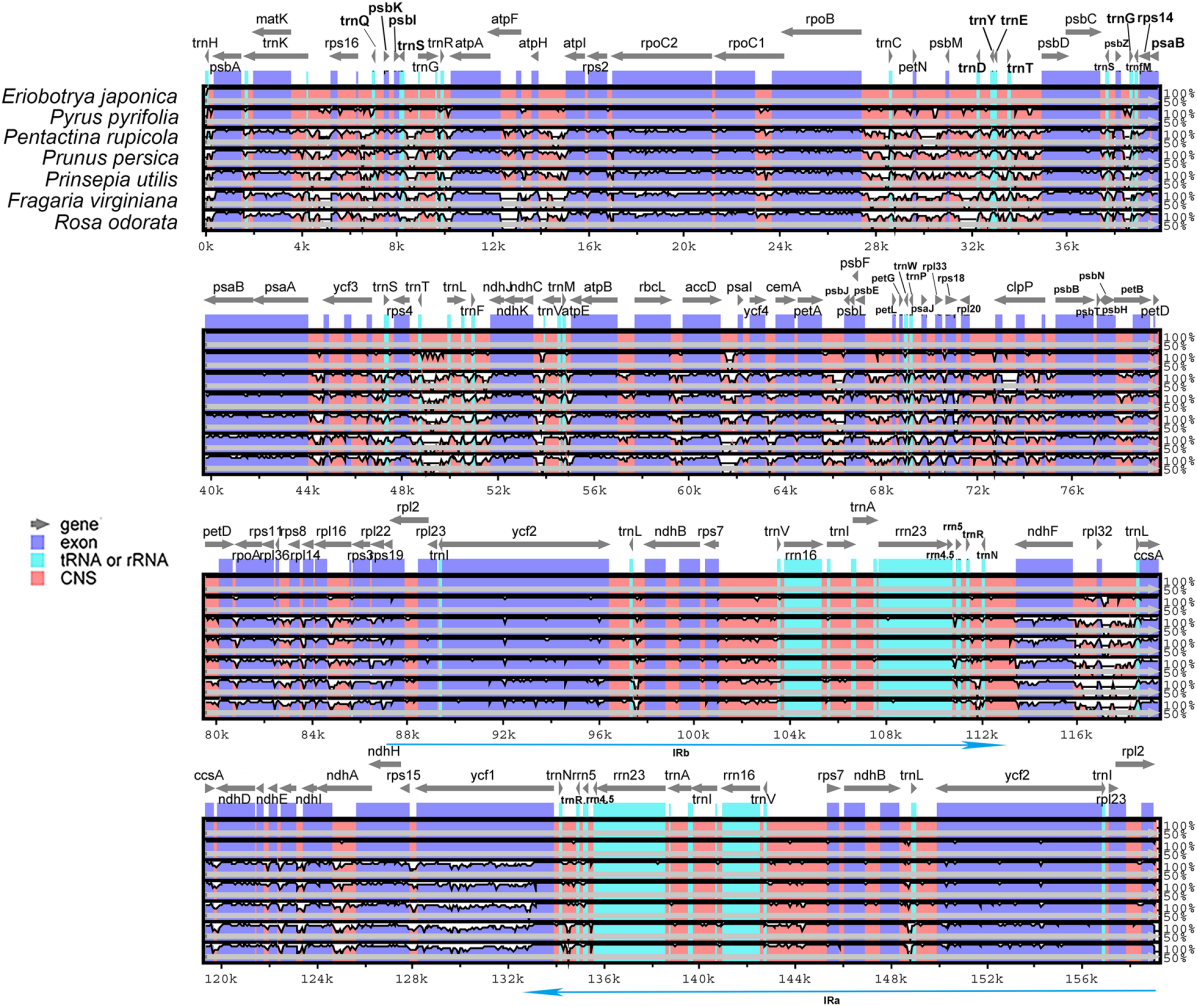
Six Rosaceae plastid genomes were aligned with *E. japonica* pairwise. Y-scale stands for identity from 50 to 100%. *Blue* represents exons of protein-coding genes, *lime* represents tRNA or rRNA genes and *red* represents non-coding regions

The multialignment of Rosaceae plastid genomes (Fig. 3) illustrated that the IR regions revealed higher identity in comparison with single copy (SC) regions. *Eriobotrya japonica* plastid genome is most similar to *P. pyrifolia* and most divergent from *Rosa odorata* and *Fragaria virginiana*. Another aspect is that coding regions show higher conservation than non-coding regions.

The GC content is similar among these species ranging from 36.7 to 37.2% as the GC content of seed plants usually ranges from 34 to 40% (Guisinger et al. 2011; Yap et al. 2015; Cai et al. 2006; Raubeson et al. 2007). *Rosa odorata* and *F. virginiana* contain the highest GC content (37.2%) while *P. pyrifolia* contains the lowest (36.6%). In addition, IR regions contain the highest GC content followed by LSC and SSC regions.

### Repeat structure

A total of 42 repeat structures containing no less than 20 bp with 100% identity were found (Additional file 3: Table S3). The number of direct, palindrome, complement and reverse structures are 29, 8, 3, 2, respectively. Of all these structures, the longest one is 59 bp located between *rpl32* and *trnL*-*UAG*. Most repeat structures located in intergenic or intron regions while two repeat structures located in *ycf1* gene.

Seventy-two simple sequence repeats including 70 mononucleotides and 2 dinucleotides were identified in *E. japonica* plastid genome (Table 4). Among 70 mononucleotides, 26 A stretches, 42 T stretches and 2 C stretches were detected while no G stretch was found. Two dinucleotide repeats, are both composed of A and T, 1 AT stretch (6 repeat motifs) and 1 TA (8 repeat motifs) stretch. The size of all SSRs are between 10 and 20 bp in length. Only five genes, *atpB*, *rpoB*, *rpoC2*, *matK* and *ycf1* appear to harbor one or two SSRs while others are all located in intergenic or intron regions. The numbers of SSRs located in LSC, IR and SSC are 58, 4, and 10, respectively.

**Table 4:** Distribution of SSRs (mononucleotide) loci in the *E. japonica* chloroplast genome

Size (bp)	Number and start position			
	A stretch	C stretch	T stretch	G stretch
10	9 (13897, 62517, 68030, 84710, 115751, 117249, 124942, 125608, 143104)	0	17 (174, 4752, 8304, 9472, 9942, 11831, 13310, 14438, 14463, 16746, 26698, 56906, 84155, 85388, 86446, 103167, 130945)	0
11	6 (16759, 44490, 46845, 51587, 68061, 158959)	0	6 (2753, 6523, 9110, 12905, 18997, 87311)	0
12	3 (187, 27779, 79320)	0	4 (1612, 51543, 67720, 85645)	0
13	2 (48460, 74027)	0	1 (71138)	0
14	1 (37903)	2 (25627, 116685)	6 (12551, 32565, 37948, 65963, 73345, 123131)	0
15	1 (6806)	0	3 (71854, 74083, 116600)	0
16	4 (69960, 80822, 131576)	0	2 (14900, 125047)	0
17	1 (7681)	0	1 (82414)	0
18	0	0	0	0
19	0	0	1 (59616)	0
20	0	0	1 (83566)	0
Total	26	2	42	0

### Nucleotide substitution and indels in *Eriobotrya japonica*, *Pyrus pyrifolia* and *Prunus persica*

**Fig. 4:**
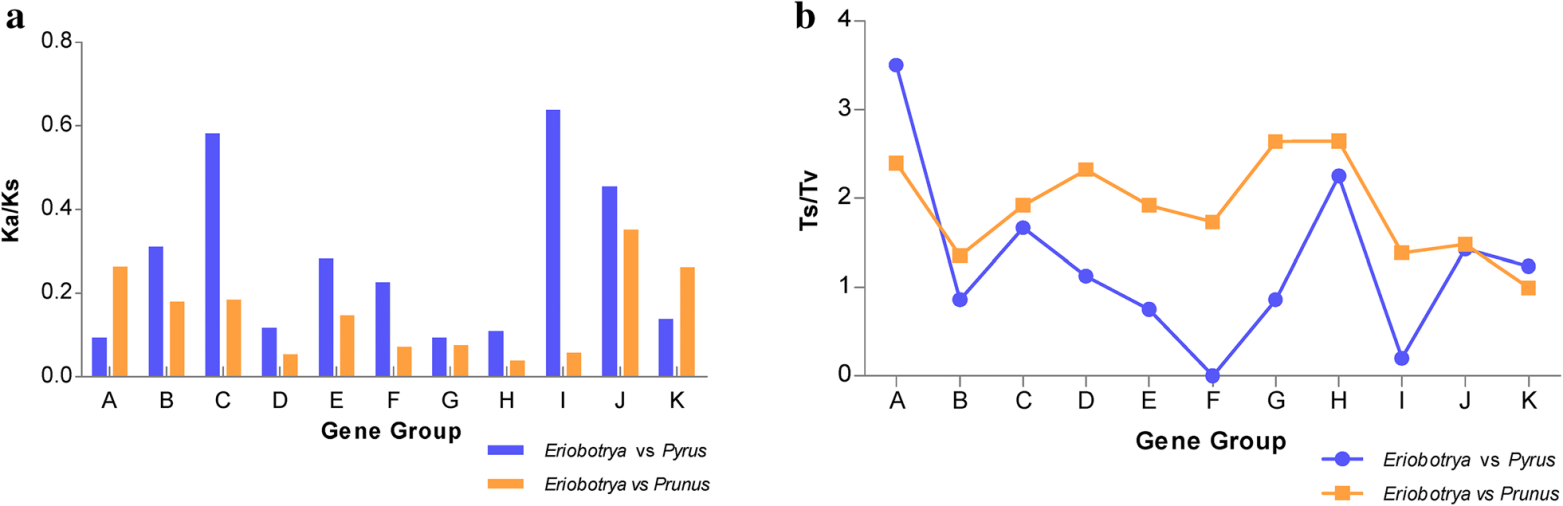
Nucleotide substitution analysis of different functional groups among *E. japonica*, *P. pyrus* and *P. persica*. Group *A* to *K* separately refers to small subunit of ribosome, large subunit of ribosome, RNA polymerase subunits, ATP synthase gene, NADH dehydrogenase, Cytochrome b/f complex, Photosystem I, Photosystem II, Large chain of Rubisco, Other genes and Unknown functions. **a** Synonymous (Ks) and nonsynonymous (Ka) ratios among three species. **b** Transtion (Ts) and transversion (Tv) ratios among three species

In this research, 78 functional protein-coding genes were classified into eleven groups according to their function, the Ka/Ks among *Pyrus pyrifolia*, *Prunus persica* and *Eriobotrya japonica* were computed. Although protein-coding regions were always conserved among closely related groups, Ka and Ks varied in different regions (Table 5) and different functions (Additional file 4: Table S4). Both Ks and Ka of all regions between *P. persica* and *E. japonica* were higher than those between *P. pyrifolia* and *E. japonica*. In two comparisons, Ka and Ks were highest in SSC regions and lowest in IR regions. When compared with *P. persica*, *psaC* revealed the highest Ks while *rps18* revealed the highest Ka and genes related with ATP synthase had the highest Ks value. When compared with *P. pyrifolia*, *rpl32* and *rpl36* revealed the highest Ks and Ka value respectively and genes of Photosystem I described the highest Ks value. The Ka/Ks values of all gene groups were less than one in both two comparisons (Fig. 4a) and only one gene *accD* contained the Ka/Ks value slightly greater than one when compared with *P. pyrifolia*.

**Table 5:** Synonymous rate, nonsynonymous rate, transition (Ts) and transversion (Tv) in LSC, IR and SSC regions among *E. japonica, P. pyrifolia* and *P. persica*

Region	*E. japonica* vs *P. pyrifolia*				*E. japonica* vs *P. persica*			
	Ks	Ka	Ts	Tv	Ks	Ka	Ts	Tv
LSC	0.0062	0.0014	209	313	0.0717	0.0091	1863	1706
IR	0.0035	0.00004	3	6	0.0110	0.0041	81	77
SSC	0.0091	0.0026	60	76	0.0942	0.0175	584	572
All	0.0053	0.0024	272	395	0.0694	0.0138	2528	2355
Ratio	Ka/Ks = 0.4528		Ts/Tv = 0.6858		Ka/Ks = 0.2126		Ts/Tv = 1.0964

A total of 675 and 5040 nucleotide substitutions were found in *E. japonica* plastid genome when compared with *P. pyrifolia* and *P. persica* respectively. The LSC region contained the most nucleotide substitutions as it’s the largest area of the genome (Table 5). Transitions and transversions in coding regions are shown in Additional file 5: Table S5. Overall, transitions occurred more than transversions when compared with *P. persica* than with *P. pyrifolia*. Also, the Ts and Tv ratio varied among different gene groups (Fig.4b). Genes related with Small subunit of ribosome and Photosystem II had a relatively higher ratio in both two comparisons. Specially, the line charts of two comparisons both illustrated similar Ts/Tv tendency, which indicated a Ts/Tv bias for different functional groups.

Large indels (>40 bp) were also identified among these three species (Table 6). Compared to *P. pyrifolia*, 11 indels were detected in *E. japonica* plastid genome comprising 7 deletions and 4 insertions. The largest indel was a 417 bp deletion located in intergenic region between *trnR*-*UCU* and *atpA*. When compared to *P. persica*, 20 indels including 8 deletions and 12 insertions were identified. All large indels were distributed in non-coding regions of LSC and SSC, while none were located in IR regions.

**Table 6:** Large indels identified among *E. japonica*, *P. pyrifolia* and *P. persica*

Type	*Eriobotrya* vs *Pyrus*			*Eriobotrya* vs *Prunus*		
	Location	Size (bp)	Repeat motifs	Location	Size (bp)	Repeat motifs
Deletion	*rps16*-*trnQ*-*UUG*	182	polyA	*rps16*-*trnQ*-*UUG*	151	
	*trnR*-*UCU*-*atpA*	417	AAT	*trnR*-*UCU*-*atpA*	40	
	*trnT*-*GGU*-*psbD*	54		*trnS*-*UGA*-*psbZ*	58	
	*psaA*-*ycf3*	52	polyA	*ndhC*-*trnV*-*UAC*	48	
	*ndhC*-*trnV*-*UAC*	56		*ndhC*-*trnV*-*UAC*	44	
	*ndhC*-*trnV*-*UAC*	48	polyT	*trnM*-*CAU*-*atpE*	45	TTTTG
	*rpl2*-*trnH*-*GUG*	50		*ccsA*-*ndhD*	50	AA
				*rpl2*-*trnH*-*GUG*	96	TA
Insertion	*petN*-*psbM*	79	TTCG	*rps16*-*trnQ*-*UUG*	138	
	*trnT*-*UGU*-*trnL*-*UAA*	42	CTCAAATATATGTTTATCAAT	*trnS*-*GCU*-*trnG*-*GCC*	151	
	*rpl32*-*trnL*-*UAG*	69		*rpoB*-*trnC*-*GCA*	47	
	*accD*-*psaI*	129	AA	*rpoB*-*trnC*-*GCA*	124	
				*psbZ*-*trnG*-*GCC*	148	
				*rps4*-*trnT*-*UGU*	48	
				*trnT*-*UGU*-*trnL*-*UAA*	42	
				*trnT*-*UGU*-*trnL*-*UAA*	78	
				*rpl33*-*rps18*	61	TTTAT
				*rps18*-*rpl20*	146	
				*ndhF*-*rpl32*	191	AATTT
				*rpl32*-*trnL*-*UAG*	59	

### IR expansion and contraction

**Fig. 5:**
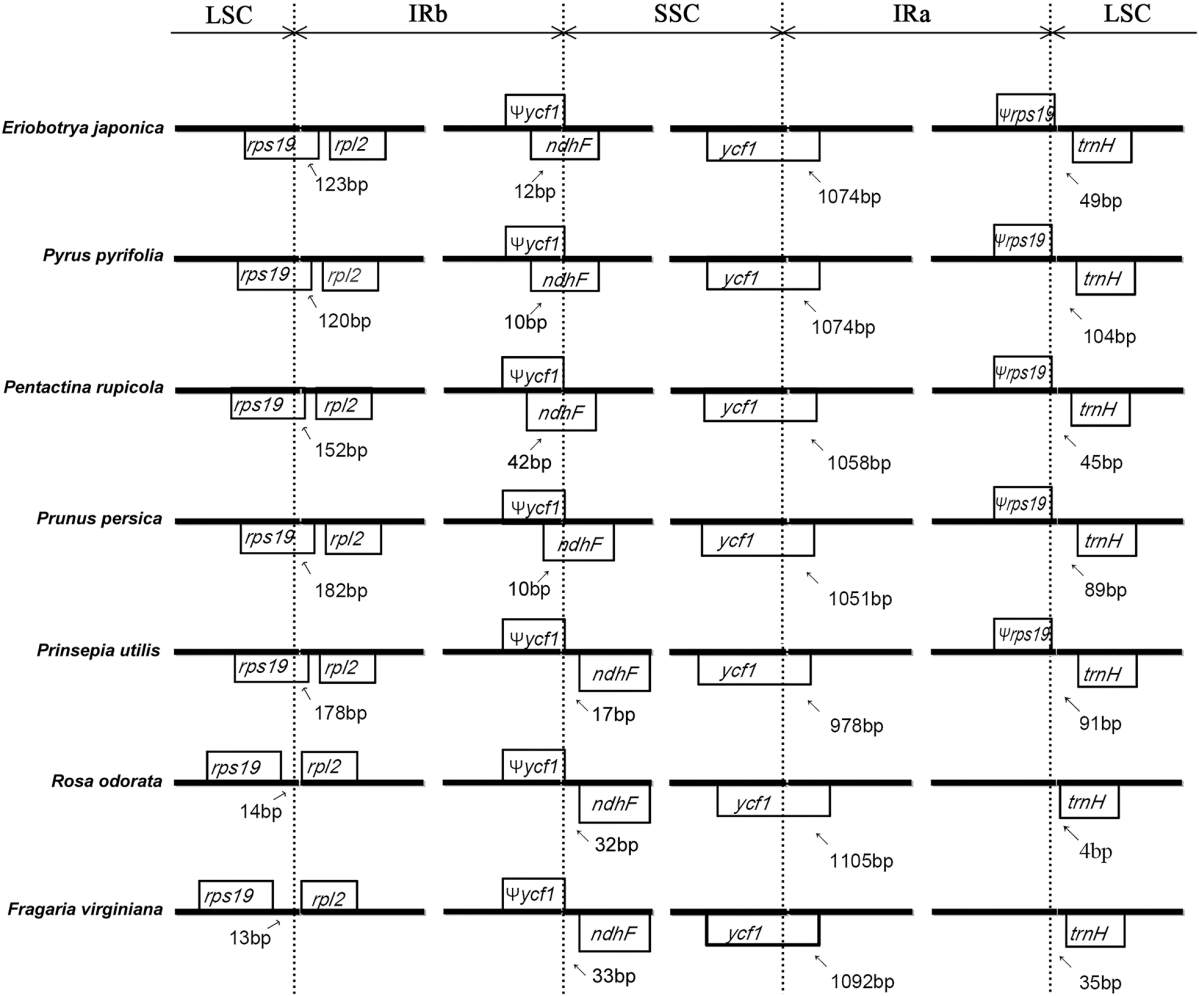
The comparison of IR boundary among 7 Rosaceae plants. Annotated genes are represented by *black boxes*. *Psi letter* means pseudogene

The SC and IR boundaries of six Rosaceae plastid genomes were aligned in Fig. 5. *Eriobotrya japonica* contained almost the same IR/SC structure with *Pyrus pyrifolia*, *Prunus persica* and *Pentactina rupicola* in which IRb/SC boundaries lay respectively in coding regions of *rps19* and *ndhF*. *Rosa odorata* and *Fragaria virginiana* contained another structure with *rps19* gene and *ndhF* gene apart from LSC/IRb boundary, which led to the loss of *rps19* pseudogene at LSC/IRa boundary. *Prinsepia utilis* contained a LSC/IRb boundary with *rps19* involved and a IRb/SSC boundary apart from *ndhF*. The IRa/SSC boundary of all six species extended to *ycf1* coding regions with the range from 978 bp (*P. utilis*) to 1105 bp (*R. odorata*), which led to a nonfunctional *ycf1* gene in IRb. The IRa/LSC boundary revealed a large variation from 4 (*R. odorata*) to 104 bp (*P. pyrifolia*) as *trnH*-*GUG* gene located upstream of IRa/LSC boundary.

### Phylogenetic analysis

All 78 protein-coding genes possessed by seven Rosaceae plastomes were extracted to perform ML and MP analysis using *Morus indica* as an outgroup.

**Fig. 6:**
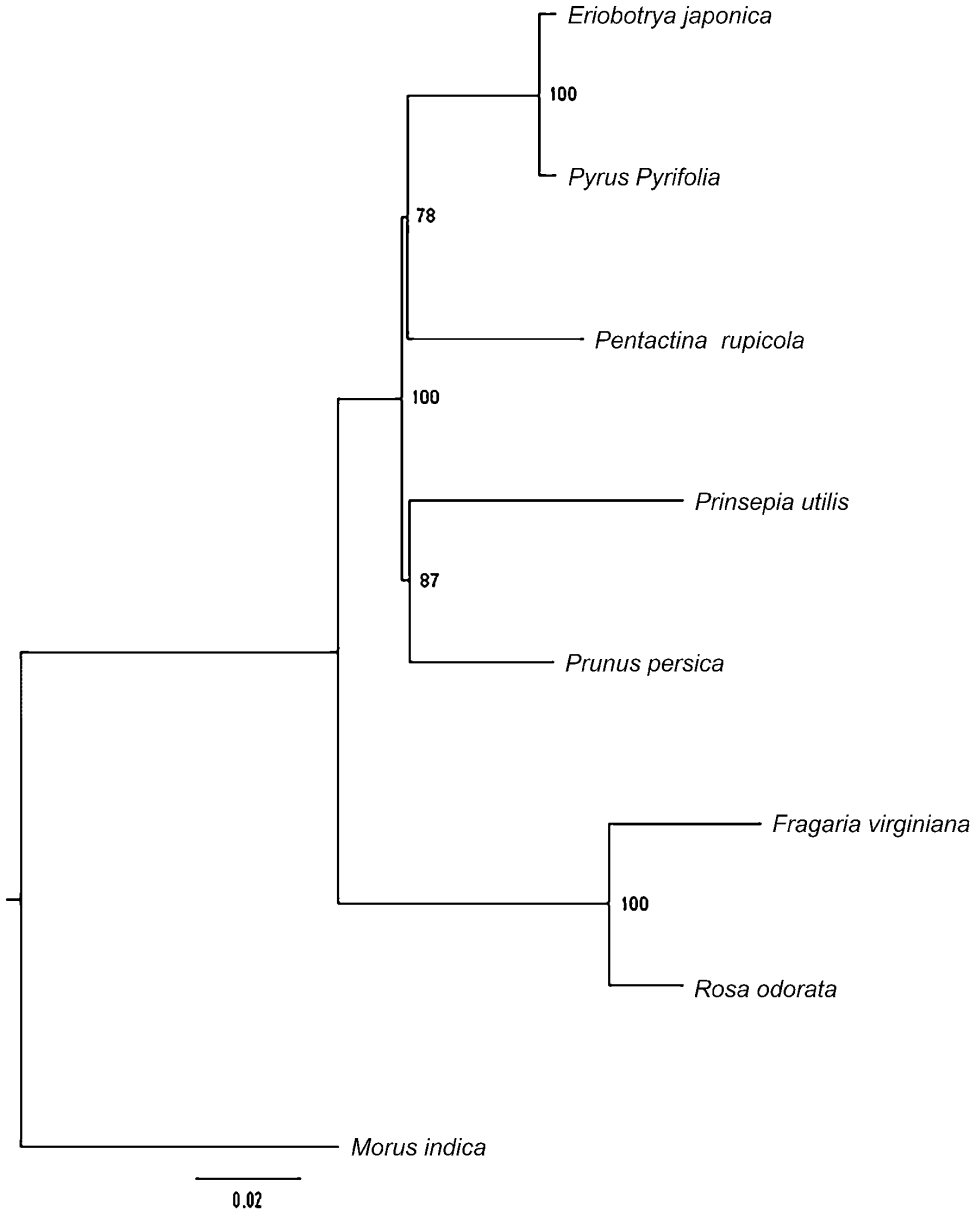
Maximum likelihood (ML) analysis using 78 protein-coding genes within Rosaceae family. *Bootstrap* values are displayed at the nodes. Length scale behind the tree indicates substitutions per site

Maximum likelihood analysis resulted in a single tree with—lnL of 150822.7449 using GTR + G + I substitution model. Maximum parsimony analysis generated one most parsimonious tree with a length of 10,628, a consistency index of 0.8852, and a retention index of 0.7117. Our MP (Additional file 6: Fig. S1) and ML (Fig. 6) trees shared similar topology. Both of them strongly supported a clade of *Fragaria viginiana* and *Rosa odorata* as sister to the remaining sampled Rosaceae species. *Prunus persica* and *Prinsepia utilis* form a clade sister to *Pentactina rupicola*, *Pyrus pyrifolia*, and *Eriobotrya japonica*. *Pyrus pyrifolia* and *Eriobotrya japonica* are sister to each other with a 100% bootstrap value.

## Discussion

We compared *Eriobotrya japonica* with available sequences of Rosaceae plastid genomes, indicating the conservation of *E. japonica* plastid genome with similar structure and gene content. However, one significant difference among these species is that *Rosa odorata* encodes *infA* gene which is non-functional in other plastid genomes due to several stop codons in coding regions (Fig. 2). It’s well recognized that plastid DNA transfers to nuclear DNA at a considerably high rate (Huang et al. 2003). However, until now, successful gene transfers to nuclear genome have been documented for only four genes in Rosids, *infA*, *rpl22*, *rpl32* and *rpoA* (Jansen et al. 2011). *infA* gene, which encodes for translation initiation factor1 was reported to transfer from plastid genome to nuclear genome for functional use several times especially in Rosids (Millen et al. 2001). So further studies could be focused on gene evolution of *infA* in Rosaceae.

Simple sequence repeats (SSRs) have been widely used as molecular markers, which is useful for plant breeding and linkage map construction. However, due to the lack of plastid genomes in Rosaceae, few chloroplast SSRs have been utilized. In this study, a total of 72 SSRs have been found. SSRs are much more abundant in non-coding regions as they always contain more mutations than conserved coding regions (Bodin et al. 2013). Of all 72 SSRs, A and T stretches accounted for 97%, which was similar with the observation in *Pyrus pyrifolia* plastid genome where 31 A stretches, 34 T stretches, 2 C stretches and no G stretch were detected (Terakami et al. 2012). These results reveal that SSR has a strong AT bias which is consistent with many studies (Ma et al. 2013; Kuang et al. 2011; Do et al. 2013).

Large indels (>40 bp) were found through the comparison among *E. japonica*, *P. pyrifolia* and *P. persica*. The distribution of large indels revealed that IR regions as well as coding regions were more conserved than other regions as none of large indels located in IR and coding regions. Many of indels were found to be flanked by short repeated motifs or polyA or T structures (Table 6), suggesting that many large indels could be attributed to slipped-strand mispairing (Levinson and Gutman 1987; Terakami et al. 2012). Five large indels were unique in *E. japonica* plastid genome including 4 deletions and 1 insertion and were located in *rps16*-*trnQ*-*UUG*, *trnR*-*UCU*-*atpA*, *ndhC*-*trnV*-*UAC*, *rpl2*-*trnH*-*GUG* and *trnT*-*UGU*-*trnL*-*UAA,* respectively. *Pentactina rupicola* plastid genome was then checked for these indels and the results showed that the deletion at *ndhC*-*trnV*-*UAC* was found in *P. rupicola*, which suggested that the other four indels might be unique to *Eriobotrya* genus.

The Ka and Ks ratio has been used to denote the rate of divergence and methods of selection pressure. The value of less than, equal to or great than 1 have been considered as purifying, neutral or positive selection, respectively (Redwan et al. 2015). *Pyrus pyrifolia* revealed a lower divergence than *P. persica* from *E. japonica* as Ka and Ks between *E. japonica* and *P. pyrifolia* were much lower (Additional file 4: Table S4). Both Ka and Ks in IR regions were much lower than those in LSC and SSC (Table 5), which suggested that IR region evolved at a slower rate than LSC and SSC (Yi and Kim 2012). The Ka and Ks ratios of all gene groups were lower than 1, suggesting that a purifying selection might act on most *E. japonica* chloroplast genes.

It has been documented that the higher Ts and Tv ratio denotes lower level of divergence and lower ratio denotes higher level of divergence (Yang and Yoder 1999; Dane et al. 2015). This might be a little incompatible with our results that Ts/Tv value is lower when compared with *P. pyrifolia* since the closest relationship between *P. pyrifolia* and *E. japonica* (Table 5). From another perspective, P-distance (nucleotide substitution per site) value was found to be much smaller in *P. pyrifolia* than in *P. persica* compared to *E. japonica* (Additional file 5: Table S5). Thus, we can infer that the lower Ts and Tv ratio between *P. pyrifolia* and *E. japonica* may be caused by extremely low Ts rate, which would then be consistent with a lower divergence between *E. japonica* and *P. pyrifolia*. If both P-distance and Ts/Tv value are considered, *ycf1* with 0.068 P-distance and 0.906 Ts/Tv value are regarded as the most divergent gene of all, which could be utilized as DNA barcode for identification.

Phylogenic analysis based on plastid genome has been rapidly developed as more and more researchers use single genes, multiple genes or different regions of plastid genome to perform phylogenetic reconstruction (Wang et al. 2013; Walker et al. 2014; Zhang et al. 2014a). Rosaceae contains nearly a hundred genera with high economic values. Yet, taxa relationship among Rosaceae has not been investigated enough and is still vague. Su et al. (2014) performed maximum likelihood analysis within Rosids using 62 conserved chloroplast genes, which suggested but with low bootstrap that *P. persica* and *P. rupicola* formed a clade while *P. pyrifolia* and *P. utilis* formed another clade. However, another ML analysis was performed using 78 protein-coding genes (Wang et al. 2013) described that *P. persica* and *P. utilis* formed a clade while *P. pyrifolia* and *P. rupicola* formed another clade. As these two studies were incongruent, we included those four genera, along with *R. odorata, F. virginiana* and *E. japonica* in our phylogenetic analysis to clarify the internal relationships within Rosaceae. Our phylogeny was similar to that of Wang et al. (2013), but with higher bootstrap values. This is also consistent with traditional studies that put *Eriobotrya* (loquat) and *Pyrus* (pear) into the subfamily Maloideae. Due to the lack of Rosaceae plastid genomes available online, in future study, more plastomes such as Spiraeae need to be involved to construct a larger-scale phylogeny within Rosaceae.

## Conclusions

The complete plastid genome of *E. japonica* is 159,137 bp in length with a typical quadripartite structure. Comparison with other Rosaceae species revealed that *E. japonica* plastid genome is quite conserved in gene content and structure and showed the highest identity with *P. pyrifolia*. A total of 72 SSRs were found including two dinucleotide repeats and most of them were composed of A/T, which showed a strong A/T bias in base composition of SSRs. Four large indels appeared to be unique to *E. japonica* and could be utilized as markers for intergeneric identification. The Ka and Ks value among three species revealed differences among different genes and Ka/Ks was always less than 1, suggesting a purifying selection on these genes. Both MP and ML phylogenetic analysis shared similar topology and strongly supported the closest relationship between *E. japonica* and *P. pyrifolia*.

## Additional files

**Additional file 1: Table S1.** Primers designed for gaps and IR/SC boundaries.

**Additional file 2: Table S2.** Primers list for InDels validation of *E. japonica*.

**Additional file 3: Table S3.** Repeat structures found in *E. japonica* chloroplast genome.

**Additional file 4: Table S4.** Synonymous (Ks) and nonsynonymous (Ka) substitution rates among *E. japonica*, *P. pyrifolia* and *P. persica*.

**Additional file 5: Table S5.** Transtions (Ts), Transversions (Tv) and P-distance among *E. japonica*, *P. persica* and *P. pyrifolia*.

**Additional file 6: Figure S1.** Maximum parsimony (MP) analysis using 78 protein-coding genes within Rosaceae family. *Bootstrap* values are displayed at the nodes.
