# Supplementary material for: Complete plastid genome of Eriobotrya japonica (Thunb.) Lindl and comparative analysis in Rosaceae
Source: Springerplus. 2016 Nov 29;5(1):2036. doi: 10.1186/s40064-016-3702-3 (PMC5127920; doi:10.1186/s40064-016-3702-3)
Supplement: Supplementary file 1 — Additional file 1: Table S1. Primers designed for gaps and IR/SC boundaries. [file 40064_2016_3702_MOESM1_ESM.doc]

**Table S1** Primers designed for gaps and IR/SC boundaries

| **No.** | **Forward** | **Reverse** | **Region** |
| --- | --- | --- | --- |
| 1 | caagctaacgatgcgggttc | agacatttaccgacgaagcg | trnS-GCU-atpA |
| 2 | gagtaacgccatggtaaggc | tgatgcaatgggttaggtcc | trnT-GGU-psbD |
| 3 | cccctcccaattccttcagg | gcgtcttctccttggcaaag | psbZ-trnG-GCC |
| 4 | gctaagcgggctcacataac | tggctataactcaccattccgt | trnT-UGU- trnL-UAA |
| 5 | ttttcgccgcacttcagatt | cgagaaggtctacggttcga | ndhC-trnV-UAC |
| 6 | ggaagggatatagggcagca | gcgatggggaaagtgataatcc | ndhF- rpl32 |
| 7 | gacgggatctcttctcatttttcg | gggttatcctgcacttggaa | LSC/IRb |
| 8 | ccgagtgaatggaaaggaaa | tggagtcggtattgcgagtt | IRb/SSC |
| 9 | tgctaaactgggtgggtacg | ccgagtgaatggaaaggaaa | SSC/IRa |
| 10 | aatttgattcttcgtcgccg | tcaaggcagtggattgtgaa | IRa/LSC |
