# Supplementary material for: Complete plastid genome of Eriobotrya japonica (Thunb.) Lindl and comparative analysis in Rosaceae
Source: Springerplus. 2016 Nov 29;5(1):2036. doi: 10.1186/s40064-016-3702-3 (PMC5127920; doi:10.1186/s40064-016-3702-3)
Supplement: Supplementary file 2 — Additional file 2: Table S2. Primers list for InDels validation of E. japonica. [file 40064_2016_3702_MOESM2_ESM.doc]

**Table S2 Primers list for InDels validation of *E. japonica***

| Indel | primer sequence (5' to 3') | | InDel size | Position |
| --- | --- | --- | --- | --- |
|  | Forward | Reverse |  |  |
| 1 | catgtccttcaagtcgcacg | aggttcgaatccttccgtcc | 182 | rps16-trnQ-UUG |
| 2 | atgcaaagcgtccattgtct | agacattttacgacgaagcgg | 417 | trnR-UCU-atpA |
| 3 | tcggggtttacagcgataact | cgttcgtatcgccggaaaag | 42 | trnT-UGU-trnL-UAA |
| 4 | attttgatcgaaccgcccag | cgagaaggtctacggttcga | 56 | ndhC-trnV-UAC |
| 5 | aatttgattcttcgtcgccg | tcaaggcagtggattgtgaa | 50 | rpl2-trnH-GUG |
