# Supplementary material for: Complete plastid genome of Eriobotrya japonica (Thunb.) Lindl and comparative analysis in Rosaceae
Source: Springerplus. 2016 Nov 29;5(1):2036. doi: 10.1186/s40064-016-3702-3 (PMC5127920; doi:10.1186/s40064-016-3702-3)
Supplement: Supplementary file 3 — Additional file 3: Table S3. Repeat structures found in E. japonica chloroplast genome. [file 40064_2016_3702_MOESM3_ESM.doc]

**Table S3** Repeat structures found in *E. japonica* chloroplast genome

| **No.** | **Size（bp）** | **Type** | **Repeat 1 Location** | **Repeat 2 Location** |
| --- | --- | --- | --- | --- |
| 1 | 59 | F | IGS(*rpl32-trnL-UAG*) | IGS(*rpl32-trnL-UAG*) |
| 2 | 42 | F | IGS(*rps12-clpP*) | IGS(rps12-clpP) |
| 3 | 40 | F | IGS(*rps12-trnV-GAC*) | intron *ndhA* |
| 4 | 40 | P | intron *ndhA* | IGS(*trnV-GAC-rps12*) |
| 5 | 31 | P | IGS(*trnM-CAU-atpE*) | IGS(*trnM-CAU-atpE*) |
| 6 | 30 | P | *trnS-GCU* | *trnS-GGA* |
| 7 | 28 | F | IGS(*rpl33-rps18*) | IGS(*rpl33-rps18*) |
| 8 | 28 | F | IGS(*rpl32-trnL-UAG*) | IGS(*rpl32-trnL-UAG*) |
| 9 | 27 | F | IGS(*rps12-clpP*) | IGS(*rps12-clpP*) |
| 10 | 27 | F | IGS(*rpl32-trnL-UAG*) | IGS(*rpl32-trnL-UAG*) |
| 11 | 27 | F | IGS(*rpl32-trnL-UAG*) | IGS(*rpl32-trnL-UAG*) |
| 12 | 26 | F | IGS(*trnC-GCA-petN*) | IGS(*trnC-GCA-petN*) |
| 13 | 24 | F | IGS(*trnT-UGU-trnL-UAA*) | IGS(*trnT-UGU-trnL-UAA*) |
| 14 | 24 | F | IGS(*trnN-GUU-ndhF*) | *ycf1* |
| 15 | 23 | F | Intron *atpF* | intron *clpP* |
| 16 | 23 | F | intron *ycf3* | IGS(*rps12-trnV-GAC*) |
| 17 | 23 | F | intron *ycf3* | intron *ndhA* |
| 18 | 23 | F | IGS(*accD-psaI*) | IGS(*accD-psaI*) |
| 19 | 23 | F | IGS(*rrn4.5-rrn5*) | IGS(*rrn4.5-rrn5*) |
| 20 | 22 | P | IGS(*psbK-psbI*) | IGS(*rbcL-accD*) |
| 21 | 22 | P | IGS(*trnR-UCU-atpA*) | IGS(*trnR-UCU-atpA*) |
| 22 | 22 | F | IGS(*atpF-atpH*) | IGS(atpF-atpH) |
| 23 | 22 | F | IGS(*rps12-clpP*) | IGS(*rps12-clpP*) |
| 24 | 22 | F | IGS(*rps12-clpP*) | IGS(*rps12-clpP*) |
| 25 | 22 | F | IGS(*rps12-clpP*) | IGS(*rps12-clpP*) |
| 26 | 22 | F | IGS(*rpl32-trnL-UAG*) | IGS(*rpl32-trnL-UAG*) |
| 27 | 22 | F | IGS(*rpl32-trnL-UAG*) | IGS(*rpl32-trnL-UAG*) |
| 28 | 21 | F | *trnS-GCU* | *trnS-UGA* |
| 29 | 21 | P | *trnS-UGA* | *trnS-GGA* |
| 30 | 21 | P | IGS(*ndhC-trnV-UAC*) | IGS(*ndhC-trnV-UAC*) |
| 31 | 21 | R | IGS(*rbcL-accD*) | IGS(*rps8-rpl14*) |
| 32 | 21 | F | IGS(*psbE-petL*) | IGS(*psbE-petL*) |
| 33 | 21 | R | IGS(*petD-rpoA*) | *ycf1* |
| 34 | 20 | P | IGS(*psbK-psbI*) | IGS(*atpH-atpI*) |
| 35 | 20 | F | IGS(*atpH-atpI*) | IGS(*rbcL-accD*) |
| 36 | 20 | C | IGS(*trnS-UGA-psbZ*) | IGS(*petA-psbJ*) |
| 37 | 20 | C | Intron *ycf3* | Intron *clpP* |
| 38 | 20 | C | IGS(*petD-rpoA*) | Intron *ndhA* |
| 39 | 20 | F | IGS(*rpl32-trnL-UAG*) | IGS(*rpl32-trnL-UAG*) |
| 40 | 20 | F | IGS(*rpl32-trnL-UAG*) | IGS(*rpl32-trnL-UAG*) |
| 41 | 20 | F | IGS(*rpl32-trnL-UAG*) | IGS(*rpl32-trnL-UAG*) |
| 42 | 20 | F | IGS(*rpl32-trnL-UAG*) | IGS(*rpl32-trnL-UAG*) |

F=forward, P=palindrome, C=complement, R=reverse, IGS=intergenic spacer.
