# Supplementary figures and images for: Complete plastid genome of Eriobotrya japonica (Thunb.) Lindl and comparative analysis in Rosaceae
Source: Springerplus. 2016 Nov 29;5(1):2036. doi: 10.1186/s40064-016-3702-3 (PMC5127920; doi:10.1186/s40064-016-3702-3)

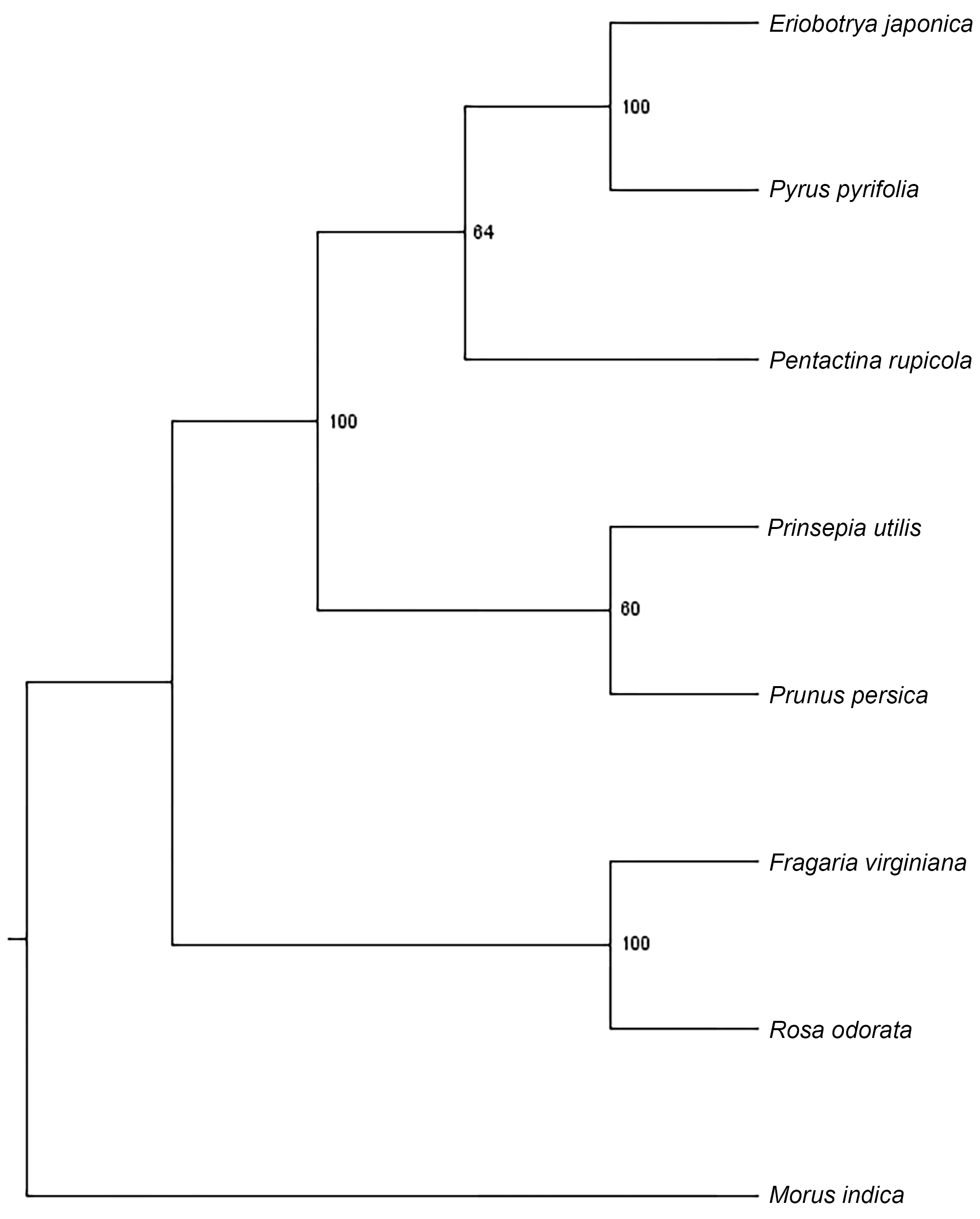

Supplement: Supplementary file 6 — Additional file 6: Figure S1. Maximum parsimony (MP) analysis using 78 protein-coding genes within Rosaceae family. Bootstrap values are displayed at the nodes. [file 40064_2016_3702_MOESM6_ESM.tif]
